# Spatial modeling of cutaneous leishmaniasis in Iranian army units during 2014-2017 using a hierarchical Bayesian method and the spatial scan statistic

**DOI:** 10.4178/epih.e2018032

**Published:** 2018-07-13

**Authors:** Erfan Ayubi, Mohammad Barati, Arasb Dabbagh Moghaddam, Ali Reza Khoshdel

**Affiliations:** 1 Modern Epidemiology Research Center, AJA University of Medical Sciences, Tehran, Iran; 2 Infectious Diseases Research Center, AJA University of Medical Sciences, Tehran, Iran; 3 Department of Preventive Medicine, Deputy of Health, AJA University of Medical Sciences, Tehran, Iran

**Keywords:** Cutaneous leishmaniasis, Military personnel, Spatial analysis, Iran

## Abstract

**OBJECTIVES:**

This study aimed to map the incidence of cutaneous leishmaniasis (CL) in Iranian army units (IAUs) and to identify possible spatial clusters.

**METHODS:**

This ecological study investigated incident cases of CL between 2014 and 2017. CL data were extracted from the CL registry maintained by the deputy of health of AJA University of Medical Sciences. The standardized incidence ratio (SIR) of CL was computed with a Besag, York, and Mollié model. The purely spatial scan statistic was employed to detect the most likely high-and low-rate clusters and to obtain the observed-to-expected (O/E) ratio for each detected cluster. The statistical significance of the clusters was assessed using the log likelihood ratio (LLR) test and Monte Carlo hypothesis testing.

**RESULTS:**

A total of 1,144 new CL cases occurred in IAUs from 2014 to 2017, with an incidence rate of 260 per 100,000. Isfahan and Khuzestan Provinces were found to have more CL cases than expected in all studied years (SIR>1), while Kermanshah, Kerman, and Fars Provinces were observed to have been high-risk areas in only some years of the study period. The most significant CL cluster was in Kermanshah Province (O/E, 67.88; LLR, 1,200.62; p<0.001), followed by clusters in Isfahan Province (O/E, 6.02; LLR, 513.24; p<0.001) and Khuzestan Province (O/E, 2.35; LLR, 73.71; p<0.001), while low-rate clusters were located in the northeast areas, including Razavi Khorasan, North Khorasan, Semnan, and Golestan Provinces (O/E, 0.03; LLR, 95.11; p<0.001).

**CONCLUSIONS:**

This study identified high-risk areas for CL. These findings have public health implications and should be considered when planning control interventions among IAUs.

## INTRODUCTION

Cutaneous leishmaniasis (CL), despite being a neglected disease, is one of the most important parasitic diseases in Iran due to several factors, including areas of high endemicity [1-3], the annual incidence of more than 24,000 cases [4], high outbreak rates [5-7], and emergence in new foci [8]. Zoonotic CL, caused by *Leishmania major*, is distributed mainly in the central [9] and southwest regions [10], while anthroponotic CL, caused by *Leishmania tropica*, affects almost all urban areas [11]. The spatial inequality of CL in Iran makes it a threat to public health and poses major challenges to control strategies [12]. Studying the spatial and geographic patterns of CL is important because the components of the chain of infection, including the parasite, host vector, and required environmental conditions, are spatially distributed [13-15]. Although several studies have been published on the epidemiological and spatial patterns of leishmaniasis in the general population in Iran, the spatial distribution of CL among special populations, such as the army and military personnel, has not been adequately studied [16,17]. The army is the most vulnerable group, with the highest incidence of this disease, due to its distinct spatial status [18], and immunologically naive troops are sometimes deployed to endemic areas for training or operational activities [19,20]. Knowledge of the spatial patterns of CL in at-risk populations can help direct control strategies for truly needy areas and reduce the burden of the disease.

When studying the spatial distribution of a disease on a defined map, however, there are some methodological challenges that must be addressed. Overlooking the effect of spatial aggregation when attempting to detect spatial clusters can increase the risk of false detection [21]. Kulldorff et al. [22]’s spatial scan statistic was introduced as an efficient method to detect spatial clusters and disease outbreaks. There is degree of population heterogeneity in the various areas of a defined map, and estimating the rate of a disease in low-population areas is accompanied by a degree of uncertainty, a phenomenon known as small area estimation or the sparse data bias; however, the rates in various areas have a degree of spatial autocorrelation, and overlooking this in spatial modeling can also induce serious bias [23-25]. Spatial methods, such as the Besag, York, and Mollié (BYM) spatial model [25,26], have been developed to address these challenges. Therefore, using up-to-date data on CL incidence from 2015 to 2017 in Iranian army units (IAUs), the objectives of the present study were to investigate the spatial distribution of CL using a BYM model and to detect significant spatial clusters of CL by applying Kulldorff et al. [22]’s spatial scan statistic.

## MATERIALS AND METHODS

### Study area and setting

Iran is situated in southwestern Asia and is the second largest country in the Middle East, with an area of 1,648,195 km². The country is located between the Caspian Sea in the north and the Persian Gulf and Gulf of Oman in the south. Iran is bordered by Afghanistan, Armenia, Azerbaijan, Iraq, Pakistan, Turkey, and Turkmenistan. The latitude and longitude of Iran are 32.4279°N and 53.6880°E, respectively. Iran has great diversity in topographical features, with elevation extremes ranging from -28 m at the Caspian Sea to 5,610 m at Mount Damavand. The great variations in altitude result in a correspondingly great diversity of environmental conditions, geographical factors, and climate indicators. In Iran, on some days temperatures can easily reach to lower than -30°C and some days reach to more than 40°C; January is the coldest month with a mean temperature of 5 to 10°C and August is the hottest month with mean temperatures of 20 to 30°C. Rainfall has a seasonal pattern, with great variation in the amount of rainfall (e.g., the mean annual rainfall ranges from 100 to 2,000 mm) [27].

### Study design and data source

An ecological study was conducted. The study included confirmed cases of CL registered by the deputy of health of AJA University of Medical Sciences between March 2014 and March 2017. The surveillance system for diseases in army units involves the systematic collection, analysis, and interpretation of health-related outcomes for use in planning, implementing, and evaluating public health and prevention interventions. This surveillance system provides a capacity for the timely referral of confirmed cases to the next higher level and finally to the deputy of health of AJA University of Medical Sciences. Reporting systems vary depending on the type of health-related outcome data and information being reported; for example, confirmed CL cases are reported monthly. The data is registered for each Anno Hegirae Solaris (A.H.S.) or solar Hijri year. A.H.S. is the unit of years used in the official calendar of Iran, with 12 months that roughly range from April of one year to March of the following year. Therefore, each year in this study was defined from April of one year to March of the following year (e.g., April 2014 to March 2015).

The data were extracted from regular surveillance reports on CL cases and transferred to Microsoft Excel (Microsoft, Redmond, WA, USA). Then, the data were entered into the Iran ArcGIS shapefile map layer for spatial analysis and to generate spatial visualizations. A queen spatial weight matrix (first order) was defined using GeoDa [28] for calculating spatial autocorrelation and for hierarchical Bayesian analysis. Weighting based on the queen order indicates whether spatial units share a boundary, so that all spatial units in contact with spatial unit *i* are considered as its neighbors.

### Statistical analysis

#### Spatial autocorrelation

The global spatial autocorrelation of CL incidence in IAUs at the province level during the study period was checked using the Moran’s index (MI). This index varies between -1.0 and 1.0; values near -1.0 indicate negative spatial autocorrelation (clustering of dissimilar values), while those close to 1.0 indicate positive spatial autocorrelation (clustering of similar values) and 0.0 indicates randomness. GeoDa [28] was used to calculate the MI and to test its statistical significance.

#### Spatial mapping

The crude incidence rate for area *i* can be calculated through dividing the number of new cases by the number of people residing in area *i*. An important goal of spatial epidemiology is to compare the incidence rates of a disease in different areas. However, the crude incidence rate does not allow valid and robust comparative inferences across areas because it is not corrected for the number of people at risk or the sex and age structure of each area, and it faces several other methodological issues in a spatial setting. In order to derive valid inferences, the incidence rates must be standardized, and indicators such as the standardized incidence ratio (SIR) must be estimated [29]. In the present study, the incidence rate was corrected only for the population at risk. Since the number of cases in each area follows a Poisson distribution, the SIR is calculated as the ratio of the observed number of incident cases of a disease (*O_i_*) in the study area *i* to the number of cases that would be expected (*E_i_*) if the study area *i* had the standard or total incidence rate. *E_i_* is calculated as follows:

$$E_{i}\;=\;n_{i}\left(\frac{\sum_{i0i}}{\sum_{ini}}\right),i\;=\;1,2,\;...\;.\;I$$

Where *n_i_* is the number of at-risk individuals in area i. Thus, the SIR is calculated as follows:

$$SIR_{i}\;=\;\frac{O_{i}}{E_{i}}$$

Crude SIRs are not corrected for spatial autocorrelation or small area estimation. Ignoring these methodological issues can result in SIRs that are biased due to overdispersion or extra-Poisson variation [24]. To obtain maps that remove the sources of overdispersion, hierarchical Bayesian approaches, such as the BYM model, have been introduced [26]. The BYM model and how the prior and hyperprior are defined for the parameters of interest have been explained in detail elsewhere [30]. Briefly, the smoothed SIRs (posterior distribution of the parameters) in a BYM model are calculated by combining the predefined information about sources of overdispersion (prior distribution) and the observed data. Sources of overdispersion are explained in the BYM model through 2 random effects: (1) the exchangeable (non-spatial) random effect *u_i_* , which refers to how the SIR in area *i* is shrunk toward the global mean of the study area (the prior distribution of *u_i_* follows a normal distribution, $u_{i}\sim N\left(0,\;\tau_{u}^{2}\right)$; and (2) the spatial random effect vi , which refers to how the SIR in area i is shrunk towards the local mean of neighboring areas and is defined by a conditional autoregressive model $N\left(\bar{\nu_{i}},\;\tau_{i}^{2}\right)$. *τ*_u_ and *τ*_v_ are the parameters of precision, and the hyperprior will be defined for them in the next step. We used a gamma (0.01, 0.01) hyperprior for $\tau_{u}^{2}$, a non-informative prior. It has been suggested that this choice involves an empirical trade-off between (a) not using a more than sufficiently informative prior when there is no robust knowledge about the spatial pattern of the outcome (e.g., CL among IAUs) and (b) enough assurance for convergence of the Markov-chain Monte Carlo (MCMC) algorithm when a flat prior is used [31]. For the spatial random effect, a gamma (0.5, 0.005) hyperprior, which is a diffuse prior, was used for $\tau_{v}^{2}$. It has been proven that with these values, an artificial spatial structure will not be imposed on the estimates of relative risks (RRs)[31].

For this study, the posterior estimates of the SIRs were obtained by simulating from the joint posterior by means of 50,000 MCMC iterations on 2 parallel chains, with the initial 4,999 discarded in the burn-in phase. The MCMC convergence was checked in several ways. First, the Brooks-Gelman-Rubin (BGR) diagnostic was used, as values of BGR near 1 indicate the convergence of the model [32]. Second, the integrated autocorrelation time was used to quantify the effects of sampling error on the results to determine whether the samples in the chain were independent. The integrated autocorrelation time directly quantifies the Monte Carlo (MC) error. As rule of thumb, an MC error lower than 5% of the posterior standard deviation (SD) guarantee convergence. Thinning was used to reduce autocorrelation in the samples in the chains, as some samples were discarded from the simulation [33]. We specified thin= 1 so that the first parameter value would be retained. We used the open-access software OpenBUGS 3.2.3 (http://openbugs.net/w/FrontPage) to conduct hierarchical Bayesian analysis. The posterior effect size estimate, median SIR, and Bayesian 95% credible intervals (2.5th and 97.5th sample percentiles) were reported. The code for the BYM model is presented in Supplementary Material 1.

#### Spatial cluster analysis

Kulldorff et al. [22]’s purely spatial scan statistic was used to identify spatial clusters with significantly high and low rates. A high-rate cluster was defined as an area where the observed number of CL cases exceeded the expected number, and a low-rate cluster was defined as an area where the observed number was lower than the expected number of cases. The spatial scan statistic imposes thousands or millions of overlapping circular windows to scan the study area. The windows (potential clusters) are centered in each study region. The radius of the window varies continuously in size from zero to a specified upper limit. By default, the maximum spatial window size (MSWS) involved 50% or less of the total population at risk. Often, the set of potential clusters is identified hierarchically, with no geographical overlap. However, the results obtained when using the hierarchical approach to define a set of non-overlapping clusters can be misleading. When the MSWS is set to 50% of the population, the hierarchical no-geographical-overlap approach provides overly large clusters with relatively small RR values, whereas when the upper limits of the MSWS are set with small sizes (e.g., 2%), small clusters with unexpectedly large RRs result [34]. It has been shown that the Gini coefficient can be used to determine the optimal MSWS [34]. To create a set of potential clusters based on the Gini index, first, the MSWS is defined with different upper limits for the size of the cluster, and then the hierarchical no-geographical-overlap approach is used to create a collection of clusters for each different upper limit. After that, the Gini index is calculated for a set of non-overlapping clusters of each upper limit, and the collection that maximizes the Gini index is the optimal upper limit [34].

Since the number of CL cases in each potential cluster has a Poisson distribution, the exponential model-based spatial scan statistic was used as a probability model to perform cluster analysis and testing. For each circular window (identified potential cluster), the likelihood ratio statistic under the Poisson distribution was calculated, as follows:

$${\left(\frac{c}{E\left[c\right]}\right)}^{c}{\left(\frac{C-c}{C-E\left[c\right]}\right)}^{C-c}$$

Where *C* is the total number of CL cases, *c* is the observed number of CL cases within the window, *E[c]* is the crude expected number of CL cases within the window under the null hypothesis, and *C-E[c]* is the expected number of CL cases outside the window. The cluster with the maximum log likelihood ratio was taken as the most likely cluster. The maximum likelihood method test was used for deviations from the null hypothesis that the number of CL cases inside and outside of the window would be equal (*H_o_: θ_in_=θ_out_* vs. the alternative hypothesis *H_a_: θ_in_≠θ_out_*). The statistical significance of the detected clusters was tested using MC hypothesis testing with 999 permutations. SaTScan version 9.4.2 (https://www.satscan.org/) developed by Kulldorff [35] was used for spatial cluster analysis. All cartographic manipulations and displays were performed in ArcGIS version 10.3 (Esri, Redlands, CA, USA).

## RESULTS

In total, 1,144 cases of CL were identified, with an incidence rate of 260 per 100,000 (86.66 per 100,000 in each year) during the study period. The MI for CL incidence showed a degree of negative spatial autocorrelation, although it was non-significant (MI, -0.12; p= 0.20 for 999 permutations).

The results of the crude SIRs (a frequentist analysis), as well as estimates from the BYM model (a Bayesian analysis), are presented in detail in Supplementary Materials 2-5. As expected, the absence of any CL cases in some provinces resulted in a crude SIR of 0; however, by borrowing information from neighbors, the BYM model provided smoothed results and the SIRs of 0 disappeared. There was little difference between the SIRs estimated in the BYM model and the crude SIRs. The value of MC error (less than 5% of posterior SD) for the estimated smoothed SIRs indicated convergence in the MCMC algorithm.

**Figure 1.**
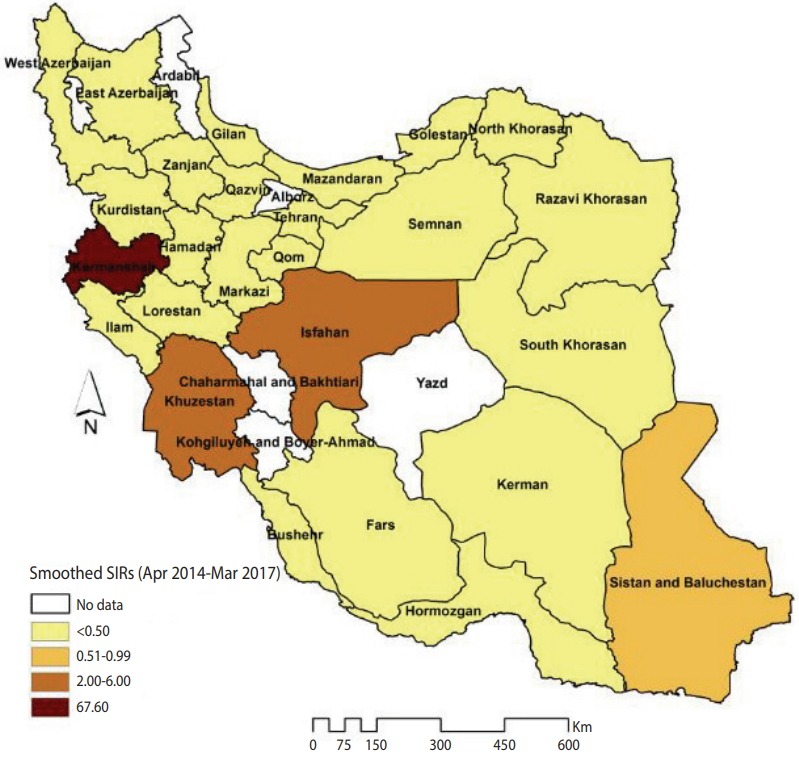
The smoothed standardized incidence ratios (SIRs) of cutaneous leishmaniasis in Iranian army units based on a Besag, York, and Mollié model (2014-2017).

**Figure 2.**
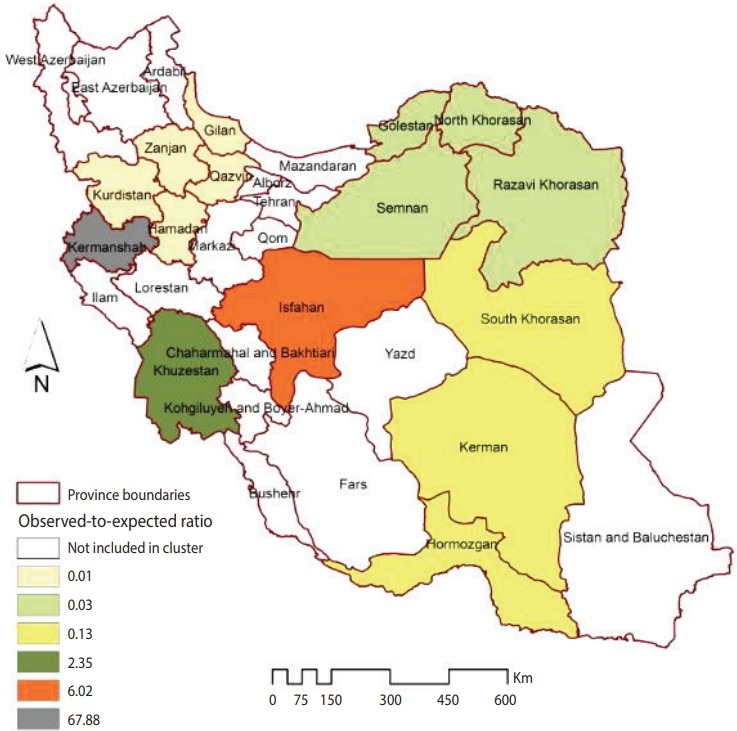
Clusters with a statistically significantly higher or lower than expected incidence of cutaneous leishmaniasis in Iranian army units (2014-2017).

Figure 1 illustrates the smoothed SIRs from the BYM model for the time period of 2014 to 2017. Figure 1 suggests that the incidence of CL was highest in the provinces of Kermanshah (SIR, 67.60), Isfahan (SIR, 6.00), and Khuzestan (SIR, 2.34). Figure 2 illustrates the results of the SaTScan spatial analysis for the most likely cluster and secondary clusters 1 and 2 for the time period of 2014 to 2017. The results of the SaTScan spatial analysis are presented in detail in Table 1. The Gini index of 0.84 indicates great statistical dispersion among the detected clusters. The maximum size cluster was 10%. A total of 10 non-overlapping SaTScan highrate and low-rate clusters were identified, only 1 of which was not a statistically significant circular window. The SaTScan spatial analysis revealed that the most likely primary spatial high-rate cluster (observed to expected [O/E], 67.88; LRR, 1,200.62; p< 0.001) was in Kermanshah Province, implying that the incidence of CL was 67.88 times greater within this cluster than in the rest of the study area during the study period. The most likely low-rate cluster (O/E, 0.03; LRR, 95.11; p< 0.001) included Razavi Khorasan, North Khorasan, Semnan, and Golestan Provinces, implying that the incidence of CL was 33.33 times lower within this cluster than in the rest of the study area.

**Figure 3.**
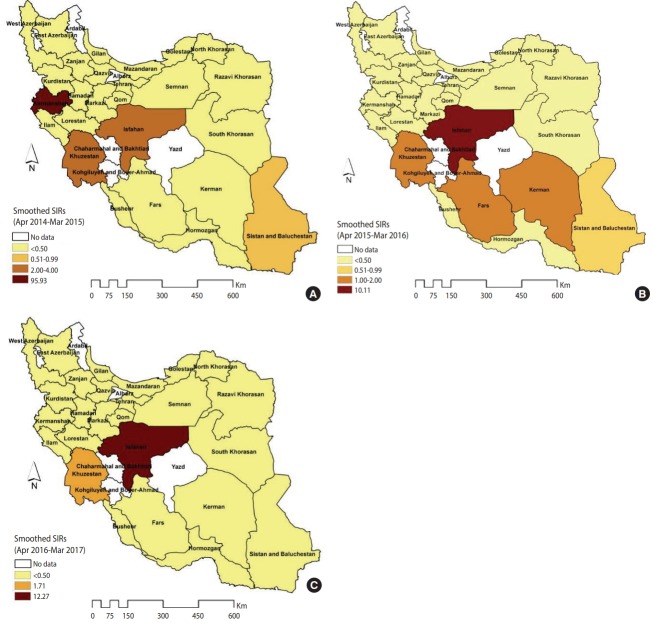
The smoothed standardized incidence ratios (SIRs) of cutaneous leishmaniasis in Iranian army units based on a Besag, York, and Mollié model (A) 2014-2015, (B) 2015-2016, and (C) 2016-2017.

The spatial patterns of the smoothed SIRs from the BYM model in the 3 distinct time periods of 2014-2015, 2015-2016 and 2016-2017 are shown in Figure 3. The spatial pattern of the most likely high-rate and low-rate clusters from the SaTScan spatial analysis for the 3 time periods of 2014-2015, 2015-2016, and 2016-2017 are shown in Supplementary Material 6. The estimation performance of the BYM model and the SaTScan spatial analysis was similar. In 2014-2015, the highest rates occurred in Kermanshah Province (SIR, 95.93), followed by Isfahan and Khuzestan Province (SIR, 2.00-4.00) (Figure 3A). In 2015-2016, the number of CL cases exceeded the expected values in the 4 provinces of Isfahan (SIR, 10.11), Khuzestan (SIR, 1.98), Kerman (SIR, 1.75), and Fars (SIR, 1.12) (Figure 3B). However, in 2016- 2017, only 2 provinces (Isfahan and Khuzestan) were at an elevated risk for CL (e.g., the number of CL cases in Isfahan Province was 12.27 times higher than was expected) (Figure 3C).

## DISCUSSION

Spatial analysis at different geographical levels can provide valuable information for developing policies to support the equitable distribution of healthcare and prevention resources. This study aimed to explore and map the spatial distribution of CL in IAUs, with cluster detection of CL incidence at the province level. The incidence of CL was 260 per 100,000 among IAUs during 2014- 2017 (86.66 per 100,000 in each year). A hierarchical Bayesian analysis showed a degree of inequality in the spatial distribution of CL, as the provinces of Isfahan, Khuzestan, Kermanshah, Kerman, and Fars had a higher than expected risk. The spatial scan statistic identified significant clusters that indicated that the most likely clusters of CL were primarily located in Kermanshah, Isfahan, and Khuzestan Provinces. In contrast, the most significant low-rate clusters of CL were identified in the northeastern provinces of Razavi Khorasan, North Khorasan, Golestan, and Semnan.

In the present study, the annual incidence rate of CL in IAUs was found to be 86.66 per 100,000, which is in accordance with previous studies that have shown the incidence of CL in the military population to be higher than its incidence in the general population in Iran [16,36]. For example, the study of Holakouie-Naieni et al. [36], which aimed to conduct spatial modeling of CL in Iran from 1983 to 2013, showed that the annual incidence of CL was 30.90 per 100,000 in the Iranian population. The study of Pakzad et al. [16], which compared the incidence of CL between IAUs and the general population during 2005-2014, showed that the incidence of CL in IAUs (143.68 per 100,000) was higher than the incidence in the general population (25.86 per 100,000). Based on a comparison with the study of Pakzad et al. [16] it can be deduced that a considerable decline in CL incidence had occurred among IAUs by 2014-2017.

Military forces are one of the most vulnerable groups to CL, and special attention to this group is very important for the prevention and control of this disease. The employment conditions of the armed forces are unique, in that soldiers are forced to settle in rural, border, and marginal urban areas and to live in areas where leishmaniasis is endemic; moreover, due to occasional large-scale transportation (e.g., military operations), they transfer the disease to other parts of the country [17,19,37].

Our results showed that the estimation performance of the BYM model and the Poisson model was similar. This implies that there was no underlying extra-Poisson variability in the data and that the introduction of spatial and non-spatial structured random effects in the BYM model fitted to the dataset was of little value for mapping modeling. In other words, modeling a spatial structure when it does not actually exist in the data may lead to a phenomenon known as over-fitting. The main reason for applying the BYM model in the present study was to explain all residual spatial variation, so we focused on providing robust and valid RRs. Our results showed that there was a degree of negative spatial autocorrelation in the distribution of CL among IAUs, although it was non-significant. Moreover, we considered a diffuse prior (e.g., a gamma [0.5, 0.005] hyperprior) for the precision parameter of the spatial random effect, as this choice precludes imposing an artificial spatial structure on the data. Although the differences between the SIRs from the Poisson model and those from the BYM model were negligible, explaining structured and unstructured spatial residuals with the BYM model may lead to much less bias in the SIRs. Moreover, a simulation study suggested that the results of ecological associations from the BYM model may be the least biased, even when true extra-Poisson variability does not exist in the dataset [38].

Our study showed that the provinces located in the western (Kermanshah), central (Isfahan), and south and southwestern areas (Khuzestan) were at a higher risk for CL during the study period. The studies in this field have shown that provinces located in cold regions, such as northern and northwestern Iran, mostly along the Zagros and Alborz mountains, had a lower incidence of CL. Due to the high altitude of those regions, the common vector of CL cannot transmit the pathogen. It has been concluded that the incidence of CL has an inverse correlation with altitude, because cold has a negative effect on the survival of the vector and the parasite of CL [39-41]. Similarly, other studies have shown that dry and desert areas had higher incidence rates due to having appropriate temperatures for carriers and parasites [42].

Due to variability in climactic conditions and the distinct activities of military forces, the risk of outbreaks should always be considered. The SaTScan spatial analysis showed that the most likely cluster of higher than expected incidence of CL was located in Kermanshah Province. Kermanshah Province is located in a mountainous region along the Zagros mountains in Iran, but within this province, there exists great variability in climactic and ecological conditions, as some counties (e.g., Sarpol-e Zahab and Qasr-e-Shirin) are located in arid and tropical regions. Studies have shown that the majority of CL cases in Kermanshah Province could be attributed to the 2 counties of Sarpol-e Zahab and Qasr-e-Shirin, which have warm and dry weather that provides a suitable setting for the survival of the vector and parasite of CL [43,44]. Thus, from the O/E ratio of 67.88 for the Kermanshah cluster, it can be concluded that when environmental conditions permit the chain of infection of CL to be established, outbreaks of CL can occur among army personnel to a remarkable extent.

This study also showed that secondary and tertiary high-risk clusters were located in Isfahan and Khuzestan Provinces, respectively. These findings are consistent with studies conducted in this field [16,17]. The existence of ideal environmental conditions for the vector, reservoir, and parasite of CL in these provinces has made them active foci for CL [45-47]. Additionally, inappropriate and inadequate sanitary facilities increase the chance of growth and proliferation of reservoirs (rodents) and vectors of CL in these 2 provinces. Our study has several limitations that should be considered. First, an obvious weakness of ecological studies, such as our study, is the ecological fallacy, whereby inferences on the aggregate level may not be true on the individual level. Second, the observed results may have been influenced by the modifiable areal unit problem, which is a well-known problem in geography in which results may be the function of the defined area units. Third, the estimated SIRs for CL may have been influenced by climactic variables, because the components of the chain of CL infection are all environmentally sensitive. However, data on climactic variables were not available in our study. Finally, we used a type of administrative and surveillance data, but results derived from such data should be interpreted with caution, because these data may not have been gathered for research purposes.

In conclusion, our study showed that the incidence of CL in IAUs was distributed differently at the province level. Due to the distinct conditions of army personnel, outbreaks of CL in non-endemic areas such as Kermanshah are expected. Additionally, army personnel in regions with clusters of CL, such as Isfahan and Khuzestan Provinces, must be targeted for further prevention and control interventions.

## Figures and Tables

**Table 1.** Characteristics of detected clusters with a higher or lower than expected incidence of CL in Iranian army units (2014-2017)

	Optimal Gini coefficient	MSC	Clusters detected	Involved province(s)	Observed cases	Expected cases	O/E	RR^1^	LLR	p-value
Area rate										
High	0.84	0.1	1	Kermanshah	353	5.20	67.88	97.73	1,200.62	<0.001
			2	Isfahan	454	75.40	6.02	9.33	513.24	<0.001
			3	Khuzestan	232	98.8	2.35	2.69	73.71	<0.001
Low			1	Razavi Khorasan, North Khorasan, Semnan, Golestan	3	104.00	0.03	0.03	95.11	<0.001
			2	Zanjan, Qazvin, Hamadan, Kurdistan, Gilan	1	96.20	0.01	0.01	94.83	<0.001
			3	Kerman, South Khorasan, Hormozgan	15	114.40	0.13	0.12	73.57	<0.001
			4	East Azerbaijan	13	91	0.14	0.13	55.52	<0.001
			5	Bushehr, Fars	28	104	0.27	0.25	41.97	<0.001
			6	West Azerbaijan	2	31.20	0.06	0.06	24.08	<0.001
			7	Qom, Markazi	1	7.80	0.13	0.13	4.76	0.09

CL, cutaneous leishmaniasis; MSC, maximum size cluster; O/E, observed-to-expected; RR, relative risk; LLR, log likelihood ratio.

1 RR was calculated as the observed number of cases divided by the expected number of cases within the cluster divided by the corresponding ratio outside the cluster.

## SUPPLEMENTARY MATERIALS

Supplementary Material 1: OpenBUGS code for BYM model is available at http://www.e-epih.org/.

Supplementary Material 2: Table S1 is available at http://www.e-epih.org/.

Supplementary Material 3: Table S2 is available at http://www.e-epih.org/.

Supplementary Material 4: Table S3 is available at http://www.e-epih.org/.

Supplementary Material 5: Table S4 is available at http://www.e-epih.org/.

Supplementary Material 6: Figure S1 is available at http://www.e-epih.org/.
