# Supplementary material for: Spatial modeling of cutaneous leishmaniasis in Iranian army units during 2014-2017 using a hierarchical Bayesian method and the spatial scan statistic
Source: Epidemiol Health. 2018 Jul 13;40:e2018032. doi: 10.4178/epih.e2018032 (PMC6186865; doi:10.4178/epih.e2018032)
Supplement: Supplementary file 1 [file epih-40-e2018032-supplementary1.pdf]

Supplementary Material 1

BYM modeling code

```
model {

        # Likelihood
            for (i in 1 : N) {
                O[i]  ~ dpois(mu[i])
                log(mu[i]) <- log(E[i]) + alpha0 + b[i] +h[i]
                # Area-specific relative risk (for maps)
                RR[i] <- exp(alpha0 + b[i]+h[i])

# Exchangeable prior on unstructured random effects
                h[i] ~ dnorm(0, tau.h)
                }
        # CAR prior distribution for random effects:
            b[1:N] ~ car.normal(adj[], weights[], num[], tau.b)
            for(k in 1:sumNumNeigh) {
                weights[k] <- 1
            }

        # Other priors:
            alpha0  ~ dflat()
            tau.b  ~ dgamma(0.5, 0.005)                  # prior on
precision
            sigma.b <- sqrt(1 / tau.b)              # standard deviation
            tau.h  ~ dgamma(0.01, 0.01)    # prior on precision
            sigma.h <- sqrt(1 / tau.h)      # standard deviation
        }
```
