# Supplementary material for: Spatial modeling of cutaneous leishmaniasis in Iranian army units during 2014-2017 using a hierarchical Bayesian method and the spatial scan statistic
Source: Epidemiol Health. 2018 Jul 13;40:e2018032. doi: 10.4178/epih.e2018032 (PMC6186865; doi:10.4178/epih.e2018032)
Supplement: Supplementary file 2 [file epih-40-e2018032-supplementary2.pdf]

Supplementary Material 2

Table S1. Median SIRs and the precision parameters (95% credible intervals) (2014-2017)

Province	Frequentist analysis	Bayesian analysis					
	Observed SIR	Mean	SD	MC_error	2.5%	Median	97.5%
Zanjan	0.000	0.02909	0.05231	3.60E-04	2.90E-05	0.00913	0.178
West Azerbaijan	0.064	0.0644	0.04445	1.86E-04	0.008858	0.05439	0.1765
Sistan and Baluchestan	0.678	0.6731	0.1109	3.77E-04	0.4742	0.6668	0.9087
Semnan	0.000	0.01455	0.02349	1.77E-04	2.46E-05	0.00558	0.0811
Qom	0.000	0.06369	0.1281	8.48E-04	3.92E-05	0.01573	0.4336
Qazvin	0.000	0.01206	0.01923	1.54E-04	1.84E-05	0.004623	0.06741
Mazandaran	0.000	0.01756	0.02937	2.26E-04	2.21E-05	0.006262	0.102
Markazi	0.192	0.1749	0.1728	7.16E-04	0.006959	0.1213	0.6378
Hamadan	0.000	0.01368	0.02153	1.82E-04	2.49E-05	0.005392	0.07569
Kurdistan	0.000	0.01331	0.02116	1.62E-04	2.16E-05	0.005131	0.07442
Khuzestan	2.348	2.343	0.1537	5.09E-04	2.051	2.341	2.654
Razavi Khorasan	0.035	0.03613	0.02446	1.08E-04	0.005144	0.03069	0.09795
North Khorasan	0.000	0.03857	0.07245	5.30E-04	2.52E-05	0.01081	0.25
Kermanshah	67.885	67.69	3.616	0.01192	60.78	67.6	74.98
Kerman	0.207	0.2025	0.07698	2.85E-04	0.08135	0.1928	0.3788
Ilam	0.000	0.02109	0.03553	2.77E-04	2.75E-05	0.007423	0.1232
Hormozgan	0.035	0.03657	0.02469	1.13E-04	0.00531	0.03113	0.09853
Lorestan	0.000	0.01128	0.01758	1.38E-04	2.08E-05	0.004523	0.06133
Golestan	0.048	0.05035	0.04672	2.46E-04	0.002413	0.03668	0.1754
Gilan	0.064	0.06499	0.06133	3.05E-04	0.003024	0.04687	0.2289
Fars	0.385	0.3807	0.07741	2.68E-04	0.2442	0.3755	0.5463
Isfahan	6.021	6.016	0.2832	9.64E-04	5.475	6.01	6.59

East Azerbaijan	0.143	0.1414	0.03932	1.44E-04	0.07504	0.1379	0.2281
Bushehr	0.096	0.09492	0.04723	1.86E-04	0.02651	0.08715	0.2076
Tehran	0.016	0.01693	0.007407	2.83E-05	0.005715	0.01585	0.03411
South Khorasan	0.256	0.2484	0.1018	3.75E-04	0.09159	0.2343	0.4841
alpha0		-3.039	0.7257	0.0333	-4.569	-2.995	-1.734
sigma.b		0.5865	1.547	0.06964	0.04523	0.1518	5.807
sigma.h		2.922	0.6962	0.0172	1.786	2.842	4.513

SIR, standardized incidence ratio; SD, standard deviation; MC, Monte Carlo.
