# Supplementary material for: Spatial modeling of cutaneous leishmaniasis in Iranian army units during 2014-2017 using a hierarchical Bayesian method and the spatial scan statistic
Source: Epidemiol Health. 2018 Jul 13;40:e2018032. doi: 10.4178/epih.e2018032 (PMC6186865; doi:10.4178/epih.e2018032)
Supplement: Supplementary file 3 [file epih-40-e2018032-supplementary3.pdf]

Supplementary Material 3

Table S2. Median SIRs and the precision parameters (95% credible intervals) (2014-2015)

Province	Frequentist analysis	Bayesian analysis					
	Observed SIR	Mean	SD	MC error	2.5%	Median	97.5%
Zanjan	0.000	0.02555	0.06	5.61E-04	3.58E-11	0.003345	0.1935
West Azerbaijan	0.045	0.04548	0.04427	1.94E-04	0.001628	0.03224	0.1637
Sistan and Baluchestan	0.882	0.8757	0.1509	4.95E-04	0.6051	0.8664	1.197
Semnan	0.000	0.01209	0.02597	2.67E-04	3.53E-11	0.002137	0.086
Qom	0.000	0.05634	0.1464	0.001168	2.36E-10	0.005557	0.4623
Qazvin	0.000	0.01006	0.0212	2.23E-04	4.18E-12	0.001778	0.07136
Mazandaran	0.000	0.01479	0.03278	3.27E-04	4.33E-12	0.002319	0.107
Markazi	0.272	0.2392	0.2475	0.001061	0.006816	0.161	0.9105
Hamadan	0.000	0.01152	0.0239	2.34E-04	1.66E-09	0.002134	0.08102
Kurdistan	0.000	0.01102	0.02323	2.43E-04	2.16E-11	0.001957	0.0785
Khuzestan	2.565	2.561	0.1917	6.43E-04	2.201	2.556	2.948
Razavi Khorasan	0.000	0.005351	0.01064	1.23E-04	1.74E-13	0.001096	0.03632
North Khorasan	0.000	0.03374	0.08516	7.53E-04	5.83E-14	0.003692	0.2669
Kermanshah	96.114	96.01	5.112	0.01796	86.33	95.93	106.3
Kerman	0.000	0.008422	0.01727	1.85E-04	2.35E-11	0.001617	0.05799
Ilam	0.000	0.01778	0.03916	3.71E-04	3.45E-10	0.002795	0.13
Hormozgan	0.000	0.005533	0.01089	1.24E-04	9.56E-12	0.001203	0.03742
Lorestan	0.000	0.009526	0.01977	2.08E-04	6.22E-11	0.001743	0.06688
Golestan	0.068	0.06563	0.06487	2.86E-04	0.002285	0.04576	0.2417
Gilan	0.091	0.08607	0.08509	3.40E-04	0.002886	0.06018	0.3153
Fars	0.363	0.3593	0.08999	3.06E-04	0.2053	0.3514	0.5565

Isfahan	3.699	3.693	0.2637	8.85E-04	3.196	3.687	4.23
East Azerbaijan	0.202	0.2	0.05572	2.00E-04	0.1063	0.1945	0.324
Bushehr	0.034	0.03476	0.03338	1.56E-04	0.001313	0.0249	0.1241
Tehran	0.023	0.02334	0.01032	3.90E-05	0.007668	0.02184	0.04753
South Khorasan	0.363	0.3515	0.1444	4.79E-04	0.1283	0.3314	0.689
alpha0		-4.112	1.515	0.07236	-8.225	-3.793	-2.021
sigma.b		3.239	9.296	0.4249	0.04647	0.1854	33.55
sigma.h		4.759	3.657	0.1625	2.295	3.74	16.04

SIR, standardized incidence ratio; SD, standard deviation; MC, Monte Carlo.
