# Supplementary material for: Spatial modeling of cutaneous leishmaniasis in Iranian army units during 2014-2017 using a hierarchical Bayesian method and the spatial scan statistic
Source: Epidemiol Health. 2018 Jul 13;40:e2018032. doi: 10.4178/epih.e2018032 (PMC6186865; doi:10.4178/epih.e2018032)
Supplement: Supplementary file 4 [file epih-40-e2018032-supplementary4.pdf]

Supplementary Material 4

Table S3. Median SIRs and the precision parameters (95% credible intervals) (2015-2016)

Province	Frequentist analysis	Bayesian analysis					
	Observed SIR	Mean	SD	MC error	2.5%	Median	97.5%
Zanjan	0.000	0.01857	0.1467	0.001197	0	1.06E-18	0.1568
West Azerbaijan	0.000	0.005969	0.04274	5.09E-04	0	1.91E-19	0.05645
Sistan and Baluchestan	0.577	0.5703	0.3308	0.001251	0.1162	0.5083	1.384
Semnan	0.000	0.009976	0.06779	6.85E-04	0	2.44E-15	0.1002
Qom	0.000	0.06765	0.4951	0.00239	0	2.17E-14	0.6073
Qazvin	0.000	0.004846	0.04106	5.56E-04	0	5.55E-28	0.03816
Mazandaran	0.000	0.01147	0.08056	7.72E-04	0	1.66E-17	0.1093
Markazi	0.000	0.02178	0.1956	0.001616	0	1.75E-22	0.1543
Hamadan	0.000	0.009614	0.06313	6.42E-04	0	1.10E-14	0.1001
Kurdistan	0.000	0.00539	0.04679	6.05E-04	0	9.14E-30	0.03842
Khuzestan	2.018	2.017	0.4626	0.001478	1.217	1.981	3.023
Razavi Khorasan	0.000	0.004243	0.02758	3.56E-04	0	1.48E-16	0.04443
North Khorasan	0.000	0.03084	0.226	0.001594	0	9.11E-17	0.283
Kermanshah	0.000	0.03045	0.2383	0.001627	0	5.53E-17	0.2577
Kerman	1.863	1.853	0.7615	0.00297	0.6792	1.752	3.618
Ilam	0.000	0.01519	0.1041	9.10E-04	0	1.39E-15	0.1477
Hormozgan	0.183	0.1795	0.1821	7.47E-04	0.004407	0.1227	0.6641
Lorestan	0.000	0.006504	0.04642	5.23E-04	0	7.49E-18	0.06229
Golestan	0.000	0.009059	0.0639	7.07E-04	0	1.56E-17	0.08526
Gilan	0.000	0.01061	0.08153	7.92E-04	0	9.23E-19	0.09747
Fars	1.177	1.171	0.444	0.001581	0.4683	1.115	2.188
Isfahan	10.161	10.16	1.189	0.004204	7.95	10.11	12.61
East Azerbaijan	0.000	0.002429	0.01646	2.51E-04	0	1.05E-19	0.02478
Bushehr	0.000	0.006475	0.04029	4.40E-04	0	6.52E-14	0.06881

Tehran	0.000	9.33E-04	0.005608	1.02E-04	0	2.26E-17	0.0105
South Khorasan	0.000	0.01055	0.0652	6.15E-04	0	2.12E-13	0.1124
alpha0		-12.22	16.65	0.8083	-38.66	-11.18	17.08
sigma.b		113.1	91.07	3.997	0.06292	118.1	298.9
sigma.h		76.25	55.97	2.427	3.292	72.15	196.3

SIR, standardized incidence ratio; SD, standard deviation; MC, Monte Carlo.
