# Supplementary material for: Spatial modeling of cutaneous leishmaniasis in Iranian army units during 2014-2017 using a hierarchical Bayesian method and the spatial scan statistic
Source: Epidemiol Health. 2018 Jul 13;40:e2018032. doi: 10.4178/epih.e2018032 (PMC6186865; doi:10.4178/epih.e2018032)
Supplement: Supplementary file 5 [file epih-40-e2018032-supplementary5.pdf]

Supplementary Material 5

Table S4. Median SIRs and the precision parameters (95% credible intervals) (2016-2017)

Province	Frequentist analysis	Bayesian analysis					
	Observed SIR	Mean	SD	MC error	2.5%	Median	97.5%
Zanjan	0.000	0.02079	0.09875	0.001254	0	2.22E-12	0.2232
West Azerbaijan	0.162	0.1521	0.1551	7.69E-04	0.00394	0.1036	0.5709
Sistan and Baluchestan	0.000	0.00588	0.02199	3.99E-04	0	1.78E-10	0.06254
Semnan	0.000	0.01154	0.04788	7.09E-04	0	5.91E-10	0.123
Qom	0.000	0.05391	0.2774	0.002174	0	3.17E-09	0.5681
Qazvin	0.000	0.00898	0.03726	6.28E-04	0	2.53E-12	0.09925
Mazandaran	0.000	0.01265	0.05548	8.30E-04	0	4.50E-12	0.1386
Markazi	0.000	0.0315	0.1584	0.001492	0	9.53E-10	0.327
Hamadan	0.000	0.01103	0.04388	6.47E-04	0	5.66E-09	0.1177
Kurdistan	0.000	0.008552	0.03754	6.74E-04	0	6.61E-18	0.09385
Khuzestan	1.734	1.728	0.2979	0.001132	1.191	1.711	2.356
Razavi Khorasan	0.176	0.1706	0.1222	5.57E-04	0.02072	0.1418	0.4822
North Khorasan	0.000	0.03043	0.1488	0.001565	0	9.07E-11	0.3243
Kermanshah	0.000	0.0288	0.1442	0.001534	0	4.30E-11	0.2983
Kerman	0.149	0.1423	0.1451	7.03E-04	0.003782	0.09689	0.5347
Ilam	0.000	0.01739	0.07525	9.49E-04	0	2.69E-09	0.1841
Hormozgan	0.088	0.08585	0.08584	3.71E-04	0.002401	0.05916	0.3169
Lorestan	0.000	0.008421	0.03495	5.83E-04	0	8.21E-12	0.09265
Golestan	0.000	0.01074	0.0455	7.21E-04	0	5.75E-12	0.1165
Gilan	0.000	0.01257	0.05658	8.32E-04	0	7.40E-13	0.1347
Fars	0.081	0.07896	0.07895	3.26E-04	0.00215	0.05474	0.2901

Isfahan	12.298	12.29	0.9057	0.003341	10.57	12.27	14.12
East Azerbaijan	0.000	0.003561	0.01341	2.70E-04	0	4.84E-13	0.03911
Bushehr	0.363	0.3549	0.2083	0.001034	0.07083	0.3148	0.8645
Tehran	0.000	0.00148	0.00505	1.17E-04	0	3.23E-12	0.01589
South Khorasan	0.000	0.0112	0.04498	6.70E-04	0	2.99E-09	0.118
alpha0		-11.5	6.982	0.3382	-25.36	-10.69	-2.349
sigma.b		78.3	62.46	2.804	0.06268	88.78	195
sigma.h		43.11	31.36	1.406	1.993	47.43	102.4

SIR, standardized incidence ratio; SD, standard deviation; MC, Monte Carlo.
