# Supplementary material for: Spatial modeling of cutaneous leishmaniasis in Iranian army units during 2014-2017 using a hierarchical Bayesian method and the spatial scan statistic
Source: Epidemiol Health. 2018 Jul 13;40:e2018032. doi: 10.4178/epih.e2018032 (PMC6186865; doi:10.4178/epih.e2018032)
Supplement: Supplementary file 6 [file epih-40-e2018032-supplementary6.pdf]

Supplementary Material 6

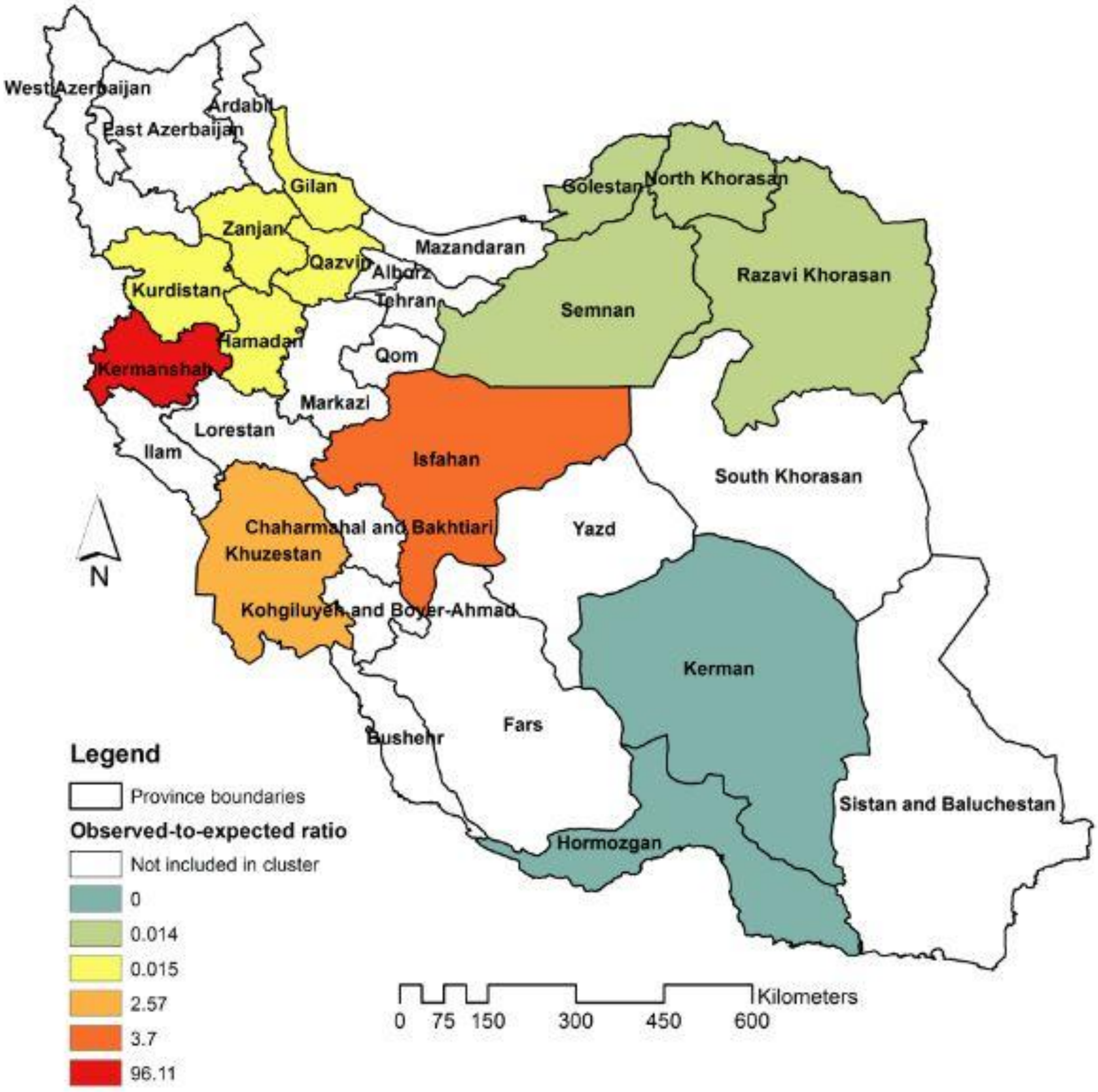

(A)

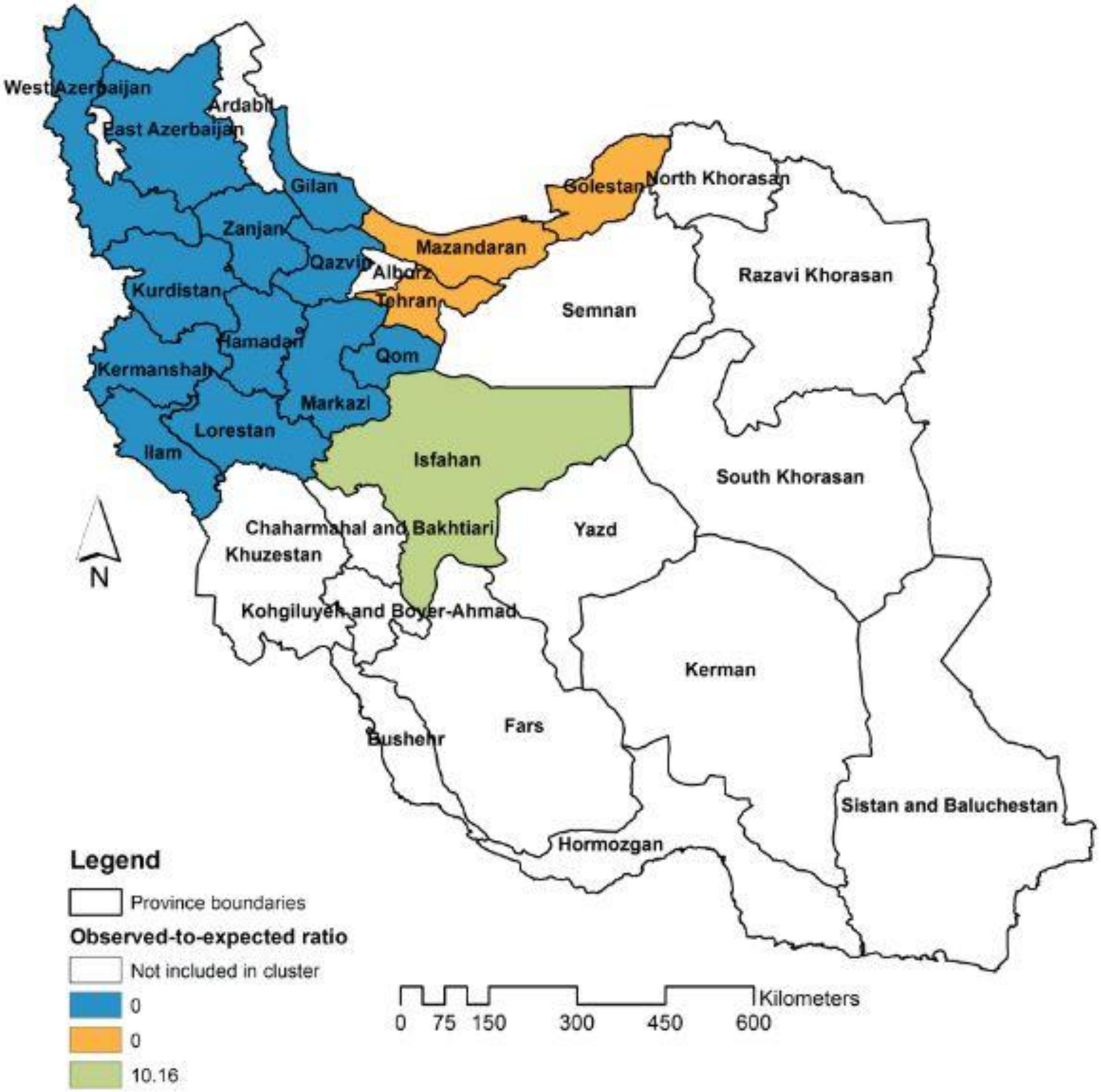
West Azerbaijan
East Azerbaijan
Ardabil
Gilan
Zanjan
Golestan
North Khorasan
Mazandaran
Qazvin
Alborz
Razavi Khorasan
Kurdistan
Tehran
Semnan
Hamadan
Qom
Kermanshah
Markazi
Lorestan
Isfahan
Ilam
South Khorasan
Chaharmahal and Bakhtiari
Yazd
Khuzestan
N
Kohgiluyeh and Boyer-Ahmad
Kerman
Fars
Bushehr
Sistan and Baluchestan
Hormozgan
Legend
Province boundaries
Observed-to-expected ratio
Not included in cluster
0
0
10.16
0 75 150 300 450 600
Kilometers

(B)

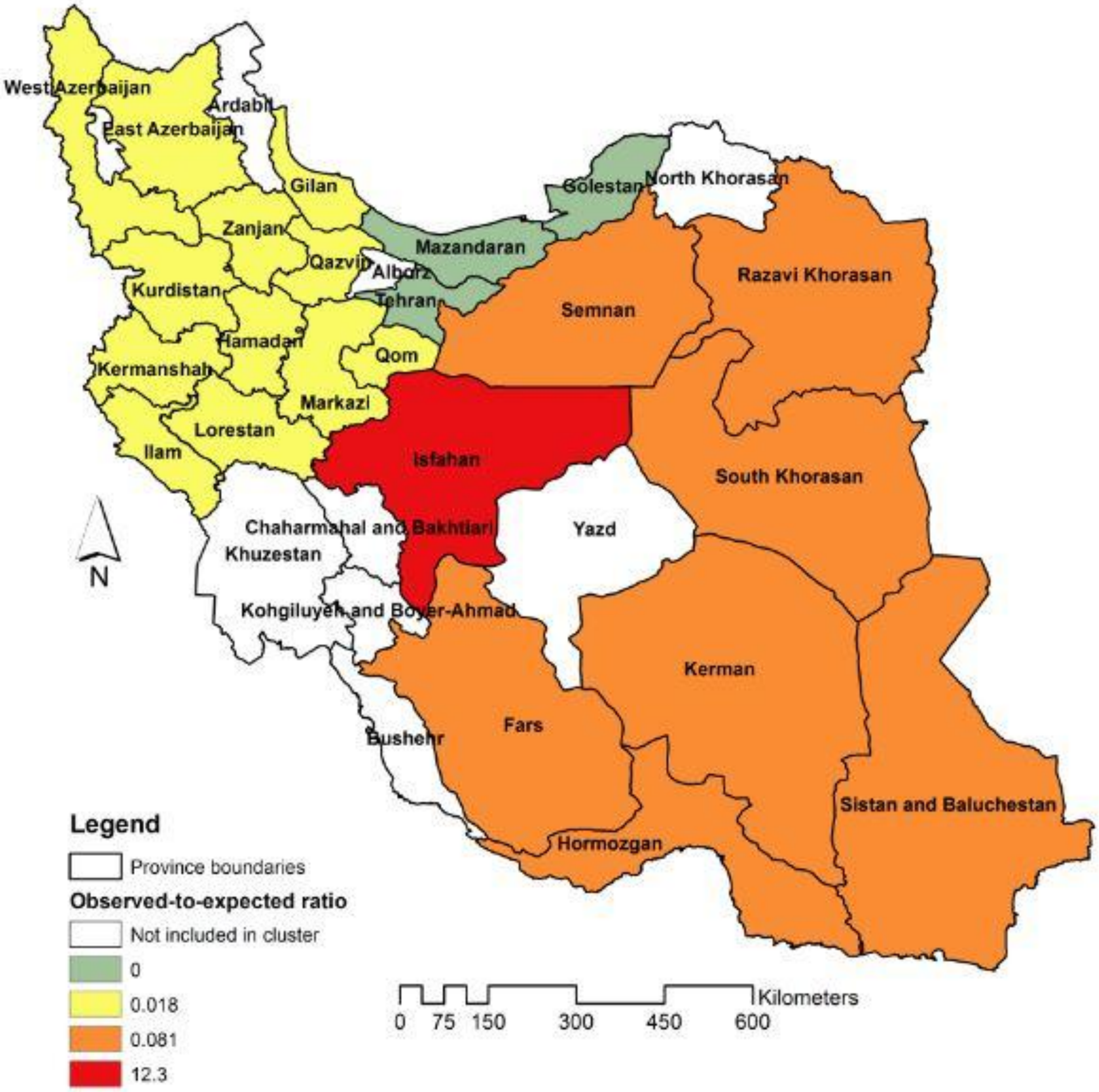

(C)

Figure S1. Clusters with statistically significant higher or lower than expected incidence of cutaneous leishmaniasis in Iranian army units (A) 2014-2015, (B) 2015-2016, and (C) 2016-2017.
